# Ovarian Cancer and Body Size: Individual Participant Meta-Analysis Including 25,157 Women with Ovarian Cancer from 47 Epidemiological Studies

**DOI:** 10.1371/journal.pmed.1001200

**Published:** 2012-04-03

**Authors:** Collaborative Group on Epidemiological Studies of Ovarian Cancer

**Affiliations:** Cancer Epidemiology Unit, University of Oxford, Oxford, United Kingdom; McGill University, Canada

## Abstract

**Background:**

Only about half the studies that have collected information on the relevance of women's height and body mass index to their risk of developing ovarian cancer have published their results, and findings are inconsistent. Here, we bring together the worldwide evidence, published and unpublished, and describe these relationships.

**Methods and Findings:**

Individual data on 25,157 women with ovarian cancer and 81,311 women without ovarian cancer from 47 epidemiological studies were collected, checked, and analysed centrally. Adjusted relative risks of ovarian cancer were calculated, by height and by body mass index.

Ovarian cancer risk increased significantly with height and with body mass index, except in studies using hospital controls. For other study designs, the relative risk of ovarian cancer per 5 cm increase in height was 1.07 (95% confidence interval [CI], 1.05–1.09; *p*<0.001); this relationship did not vary significantly by women's age, year of birth, education, age at menarche, parity, menopausal status, smoking, alcohol consumption, having had a hysterectomy, having first degree relatives with ovarian or breast cancer, use of oral contraceptives, or use of menopausal hormone therapy. For body mass index, there was significant heterogeneity (*p*<0.001) in the findings between ever-users and never-users of menopausal hormone therapy, but not by the 11 other factors listed above. The relative risk for ovarian cancer per 5 kg/m^2^ increase in body mass index was 1.10 (95% CI, 1.07–1.13; *p*<0.001) in never-users and 0.95 (95% CI, 0.92–0.99; *p* = 0.02) in ever-users of hormone therapy.

**Conclusions:**

Ovarian cancer is associated with height and, among never-users of hormone therapy, with body mass index. In high-income countries, both height and body mass index have been increasing in birth cohorts now developing the disease. If all other relevant factors had remained constant, then these increases in height and weight would be associated with a 3% increase in ovarian cancer incidence per decade.

*Please see later in the article for the Editors' Summary*

## Abstract 2

A reanalysis of published and unpublished data from epidemiological studies examines the association between height, body mass index, and the risk of developing ovarian cancer.

## Editors' Summary

**Background:**

Cancer of the ovaries, usually referred to as ovarian cancer, is the fifth leading cause of cancer death in women, and, unfortunately, symptoms (such as abdominal pain and swelling) usually occur late in the disease process; fewer than one-third of ovarian cancers are detected before they have spread outside of the ovaries. There is no definitive evidence that screening reduces mortality from ovarian cancer, and given the poor prognosis of advanced ovarian cancer, there has been much research over recent years to increase understanding of this serious condition. There are recognized risk factors that increase the chance of developing ovarian cancer, such as increasing age, having fewer children, not having used oral contraceptives, and use of menopausal hormone therapy. Age and oral contraceptive use have by far the biggest impact on ovarian cancer risk.

**Why Was This Study Done?:**

To date, there is no definitive information about the relevance of women's height, weight, and body mass index to their subsequent risk of developing ovarian cancer. There have been roughly 50 epidemiological studies of ovarian cancer, but only about half of these studies have published results on the association between body size and ovarian cancer risk, and so far, these findings have been inconsistent. Therefore, the researchers—an international collaboration of researchers studying ovarian cancer—re-analyzed the available epidemiological evidence to investigate the relationship between ovarian cancer risk and adult height, weight, and body mass index, and to examine the consistency of the findings across study designs.

**What Did the Researchers Do and Find?:**

After an extensive literature search, the researchers identified 47 eligible studies that collected individual data on women's reproductive history, use of hormonal therapies, height, weight, and/or body mass index, and in which the principal investigators of each study accepted the invitation from the researchers to be involved in the re-analysis. The researchers combined data from the different studies. To ensure that women in one study were only directly compared with controls (similar women without ovarian cancer) in the same study, all analyses were routinely stratified by study, center within study, age, parity, use of oral contraceptives, use of hormonal therapy for menopause, and menopausal status or hysterectomy.

The 47 studies were conducted in 14 countries and comprised a total of 25,157 women with ovarian cancer (mostly from Europe and North America) and 81,311 women without ovarian cancer. The researchers found a significant increase in relative risk (1.07) of ovarian cancer per 5 cm increase in height. Furthermore, this risk did not vary depending on other studied factors—age, year of birth, education, age at menarche, parity, menopausal status, smoking, alcohol consumption, having had a hysterectomy, having first degree relatives with ovarian or breast cancer, use of oral contraceptives, or use of menopausal hormone therapy. However, the researchers found that for body mass index, the risks depended on whether women had ever taken menopausal hormone therapy: the relative risk for ovarian cancer per 5 kg/m^2^ increase in body mass index was 1.10 in women who had never taken menopausal hormone therapy but was only 0.95 in women who had previously taken menopausal hormone therapy.

**What Do These Findings Mean?:**

These findings suggest that increasing height can be considered as a risk factor for ovarian cancer and that in women who have never taken menopausal hormone therapy, increased body mass index can be considered an additional risk factor. These findings have public health implications, especially in high-income countries, because the average height of women has increased by about 1 cm per decade and average body mass index has increased by about 1 kg/m^2^ per decade. The findings suggest an associated increase in ovarian cancer incidence of 3% per decade if all other factors relevant for ovarian cancer remain constant.

**Additional Information:**

Please access these web sites via the online version of this summary at http://dx.doi.org/10.1371/journal.pmed.1001200.

- The following organizations give more information on ovarian cancer which may be of use to patients: MedicineNet, the US National Cancer Institute, Ovarian Cancer National Alliance, Macmillan Cancer Support

## Introduction

About 50 epidemiological studies of ovarian cancer have collected information on the relevance of women's adult height and weight to their subsequent risk of ovarian cancer [1]–[52]. Only about half these studies have published their results on the association between body size and ovarian cancer risk, and the findings are inconsistent [22],[24],[25],[29],[33],[37]–[41],[44]–[52]. An international collaboration was set up to bring together, re-analyse, and publish the available epidemiological evidence on the association between hormonal, anthropometric, and other factors and ovarian cancer risk [53]. This report describes the relationship between ovarian cancer risk and adult height, weight, and body mass index, and examines the consistency of the findings across study designs, across subgroups of women, and by tumour histology. Data from studies that had and had not published on the association with body size are included here, as this helps avoid unduly selective emphasis on particular studies, or just on published results.

## Methods

### Identification of Studies and Collection of Data

Our collaboration began in 1998, and since then potentially eligible epidemiological studies have been sought regularly, by searches of review articles and through computer-aided literature searches in MEDLINE, EMBASE, and PubMed using combinations of the search terms “ovarian cancer”, “ovary cancer”, “height”, “body mass index”, “body size”, “anthropometr*”. To be eligible for these analyses, studies needed to have collected individual data on women's reproductive history, use of hormonal therapies, height, weight, and/or body mass index, studied at least 200 women with ovarian cancer, and published their findings before 1 January 2009. (Before 2006, studies with fewer than 200 cases of ovarian cancer had been eligible, and so there are fewer cases in some studies.) Studies that had collected relevant data but had not published on ovarian cancer and body size were sought by correspondence with colleagues, by discussions at collaborators meetings (in 2000, 2005, and 2011), and by electronic searches using the additional terms “cohort”, “prospective”, “women”, and “cancer risk”.

We identified 51 eligible studies and invited principal investigators from each study to participate in the collaboration. Investigators from just one eligible study [49] did not respond to any of our enquiries, and investigators from another study [48] wrote to say that they were unable to participate. Two other studies [50],[51] have contributed to the collaboration, but their data were not available for these analyses. Thus, data from 47 of the 51 eligible studies identified are analysed here. The implications of this are discussed later.

“Cases” are women with malignant epithelial or non-epithelial ovarian cancer and “controls” are women without ovarian cancer who had not undergone bilateral oophorectomy. Information sought from principal investigators about every control and about every case included their age, ethnic group, education, height, weight and/or body mass index, age at menarche, reproductive history, use of hormonal contraceptives, use of menopausal hormonal therapy, hysterectomy, family history of ovarian or breast cancer, and consumption of alcohol and tobacco. The information sought on these factors was for the time preceding the onset of disease for cases and for an equivalent time for controls. So that similar analytical methods could be used across studies, cohort studies were incorporated using a nested case–control design, in which up to four controls were selected at random and matched at follow-up by age of the case at entry into the cohort, age at cancer diagnosis, and, where appropriate, broad geographical region. In one cohort study “cases” were women with fatal ovarian cancer [25], whereas in the other studies “cases” were women with incident disease.

Principal investigators of 47 epidemiological studies included in the analyses [1]–[47] provided individual information on adult height, weight, and/or body mass index for cases and controls (Table 1). Body mass index was calculated as weight (in kilograms) divided by height (in metres) squared. In one case–control study [1], information was available for height but not for weight or body mass index, and in seven small case–control studies [2]–[5],[8],[9],[12], information for body mass index, but not for weight or height, was available (these seven studies had been conducted more than 30 y ago, and original data on weight and height could no longer be retrieved). Information provided by principal investigators on a woman's adult weight and height before the onset of disease was used in these analyses. If more than one value for either variable was provided for a particular study, the values used in the analyses were those that best represented the woman's height and weight at that time. For retrospective case–control studies, this was usually women's height and weight some 1–5 y before the diagnosis of ovarian cancer, and at an equivalent time for controls. For prospective studies, principal investigators generally provided information on women's height and weight recorded at the time they were recruited into the cohort. A small number of women in some of the cohorts may have already lost weight because of an as-yet-undiagnosed ovarian cancer, and so sensitivity analyses were done excluding the first 4 y of follow-up in the prospective studies. All data contributed by principal investigators were checked and collated centrally so that analyses could use definitions as similar as possible across studies. Apparent inconsistencies in the data were rectified, where possible, by correspondence with the investigators. After the records had been checked and corrected, investigators were sent summary tables and listings of the variables to be used in analyses for final confirmation.

**Table 1:** Details of studies and women included.

Study (Country) [Reference]	Number of Cases/Controls	Median Year of Diagnosis (Cases)	Median Year of Birth (Cases)	Mean Age (Cases)	Mean Height (cm)/Mean Body Mass Index (kg/m^2^) (Controls)
**Prospective studies (** ***n*** ** = 17)**					
Oxford/FPA (UK) [17]	49/196	1988	1937	48.1	161.5/22.9
BCDDP (US) [38]	353/1,381	1989	1923	65.3	162.0/25.3
Nurses' Health Study (US) [46]	677/2,710	1991	1930	58.7	163.7/25.3
Iowa Women's Health [29]	179/716	1991	1924	68.0	162.4/23.8
Radiation technologists (US) [36]	44/176	1992	1945	47.5	164.5/23.6
Netherlands Cohort (Netherlands) [28]	248/1,739	1992	1923	67.8	165.2/25.1
CNBSS (Canada) [43]	481/1,922	1993	1932	59.1	161.7/25.1
Norwegian Counties [31]	130/520	1993	1937	55.1	162.8/25.8
CPS-II Mortality (US) [25]	2,697/1,1367	1994	1923	70.3	162.9/24.8
Swedish mammography [45]	289/1,143	1996	1931	64.1	163.9/25.2
CPS-II Nutrition (US) [39]	355/1,419	1997	1929	67.8	163.8/25.6
WLH (Norway/Sweden) [32]	105/417	1998	1947	48.7	165.9/23.0
NIH-AARP (US) [44]	751/3,009	1999	1932	65.9	163.1/26.7
EPIC (eight European countries) [47]	444/1,788	2000	1935	63.7	161.3/25.6
NOWAC (Norway) [30]	102/414	2000	1940	59.8	164.8/25.0
PLCO (US) [42]	201/805	2001	1933	68.1	163.1/26.9
Million Women Study (UK) [40]	3,753/15,009	2002	1941	61.0	162.0/25.7
All prospective studies	10,858/44,731	1999	1934	64.1	162.7/25.3
**Case–control studies with population controls (** ***n*** ** = 17)**					
Casagrande/Pike (US) [2]	150/150	1974	1932	40.2	NA/22.9
Weiss (US) [11]	298/1,135	1977	1921	55.1	162.2/23.3
CASH (US) [7]	575/4,238	1981	1937	41.9	164.123.0
Whittemore (US) [9]	232/679	1984	1933	50.5	NA/24.0
Shu/Brinton (China) [13]	228/229	1985	1933	48.4	158.5/22.1
Western New York (US) [27]	122/692	1988	1930	58.4	162.9/25.6
Risch (Canada) [15]	450/564	1991	1934	56.7	162.9/24.5
Green/Purdie (Australia) [22]	784/853	1992	1935	55.1	162.0/24.2
Mosgaard (Denmark) [18]	905/1,093	1992	1943	45.9	166.5/23.7
Cramer (US) [24]	563/525	1993	1942	51.1	162.9/25.3
Riman (Sweden) [33]	807/3,875	1994	1932	61.5	163.8/25.3
German OCS [26]	281/531	1995	1937	55.2	163.9/22.1
Pike/Wu (US) [35]	477/660	1995	1939	55.5	163.6/23.4
Goodman/Wu (US) [23]	719/892	1996	1942	55.0	159.0/24.5
NISOC study (Israel) [21]	1,237/2,103	1996	1939	56.0	161.0/25.0
OVCARE (US) [41]	376/1,637	1996	1950	45.7	165.0/24.9
SHARE (US) [37]	767/1,364	1996	1943	51.6	162.5/25.9
All with population controls	8,971/21,220	1994	1939	52.7	163.2/24.3
**Case–control studies with hospital controls (** ***n*** ** = 13)**					
Byers (US) [5]	163/753	1960	1908	52.3	NA/26.4
Newhouse (UK) [1]	280/582	1973	1918	54.1	160.7/NA
McGowan (US) [3]	182/192	1975	1923	50.4	NA//23.3
Paffenbarger (US) [8]	110/480	1975	1917	55.6	NA/24.1
Hildreth/Kelsey (US) [4]	62/1,047	1978	1918	60.1	NA/24.9
Hartge (US) [12]	293/333	1979	1924	54.3	NA/23.2
Booth (UK) [10]	286/489	1980	1927	50.9	162.8/24.1
Rosenberg (US) [16]	952/3,808	1983	1935	49.5	162.8/26.6
Negri/Franceschi(Italy) [14]	957/2,478	1986	1932	53.1	160.9/23.8
PEDS (US) [20]	411/1,752	1989	1933	54.5	162.5/25.5
Tzonou/Tricopoulos (Greece) [6]	319/396	1990	1929	56.0	160.9/24.7
Negri (Italy) [19]	1026/2,398	1995	1939	54.9	161.1/25.3
Zhejiang-Curtin (China) [34]	287/652	1999	1952	46.3	158.3/22.1
All with hospital controls	5,328/15,360	1986	1932	52.7	
**All 47 studies**	**25,157/81,311**	**1994**	**1935**	**57.6**	**162.7/25.0**

NA, not available.

Information on the histological classification of the ovarian cancers was provided by principal investigators of all but seven [5],[6],[9],[14],[17],[25],[36] of the 47 participating studies and was used to categorize tumours centrally, based on the classification system of the *International Classification of Diseases for Oncology* [54]. Tumours were subdivided as epithelial or non-epithelial, and the epithelial tumours were further classified as clear cell, endometrioid, mucinous, serous, mixed, or other. Wherever possible the epithelial tumours were further subdivided into those that were of borderline malignancy or fully malignant.

### Statistical Analysis and Presentation of Results

The analytical methods were similar to those used previously [53]. Data from different studies were combined by means of the Mantel-Haenszel stratification technique, subdividing data into fine strata, each with a separate estimate of standard “observed minus expected” (*O−E*) numbers of women with ovarian cancer, together with their variances and covariances [55],[56]. Use of these simple stratified *O−E* values has the advantage of avoiding assumptions about the precise forms of any relations in the data. The stratified *O−E* values, together with their variances, were summed to yield both odds ratios (subsequently referred to as relative risks) and associated *p*-values. When only two groups were compared, relative risk estimates were obtained from the *O−E* value and its variance (var[*O−E*]) by the one-step method [55],[56], as were their standard errors and confidence intervals (CIs). (The exact formula was as follows: log relative risk = [*O−E*]/var[*O−E*]). When more than two groups were compared, variances were estimated for every group, by treating the relative risks as floating absolute risks [57]. This method does not alter relative risk estimates, but reduces the variances attributed to them (except for the baseline group, where the relative risk is defined as 1.0) and allows the relative risk estimates to be treated as approximately independent in tests of heterogeneity and trend. The group-specific variances were used to calculate group-specific CIs (g-s CIs). Use of this method enables valid comparisons between any two groups, even if neither is the baseline group. Any comparison between two relative risks must, therefore, take the variation in each group into account. Heterogeneity between relative risk estimates was assessed using chi-squared statistics.

To ensure that women in one study were compared directly only with similar women in the same study, all analyses were routinely stratified by study, by centre within study, by age (in 5-y age groups, with women aged over 90 y excluded), parity (0, 1+), use of oral contraceptives (no, for <5 y, 5+ y), ever use of hormonal therapy for the menopause (yes, no), and menopausal status or hysterectomy (pre/perimenopausal, natural menopause before age 50, natural menopause after age 50, previous hysterectomy, other). Unknowns for each stratification variable were assigned to separate strata. Analyses in relation to height were also adjusted by body mass index, and analyses in relation to body mass index and to weight were also adjusted by height; results of sensitivity analyses to assess the effect of these mutual adjustments are presented. The effect on the main findings of other potential confounding factors (year of birth, ethnic group, education, family history of ovarian or breast cancer, age at menarche, menopausal status, alcohol use, and smoking) was examined by comparing results before and after stratification for each variable, in turn, and all simultaneously.

The relative risk of ovarian cancer per 5 cm increase in height and per 5 kg/m^2^ increase in body mass index was estimated by fitting a log-linear trend across categories of height (<155, 155–, 160–, 165–, 170–, 175+ cm) and body mass index (<20, 20–, 22.5–, 25–, 27.5–, 30–, 32.5–, 35+ kg/m^2^) using the median value within each category.

Results in the figures are presented by squares and lines, respectively, representing the relative risks and their corresponding 95% or 99% CIs or g-s CIs. The position of the square indicates the value of the relative risk, and its area is inversely proportional to the variance of the logarithm of the relative risk, thereby providing an indication of the amount of statistical information available for that particular estimate. When results from many studies or many subgroups are presented in the figures, 99% CIs/g-s CIs are given, since because of the multiple testing, *p*-values greater than 0.01 may well be due to chance. In the text and in figures summarizing the main results, 95% CIs are given.

In high-income countries the average height has increased by about 1 cm per decade [58], and the average body mass index has increased by about 1 unit per decade [59]. To illustrate the public health consequences of the secular trend of increasing height and weight among women in such countries, we applied the relative risks obtained here per centimetre increase in height and per unit increase in body mass index to estimate how ovarian cancer rates would have changed per decade, had all other factors relevant for ovarian cancer remained constant. The PRISMA checklist is provided as Text S1.

## Results

Details of the women in the 47 participating studies are shown in Table 1. The studies are grouped by their design and, within each type of design, are ordered by the median year when the ovarian cancers were diagnosed. Altogether the 47 studies were conducted in 14 countries. The studies contributed a total of 25,157 women with ovarian cancer (cases) and 81,311 women without ovarian cancer (controls), with almost half the cases from Europe and half from North America. The cancers were diagnosed in 1994, on average, and the mean age at diagnosis was 57.6 (standard deviation, 12.5) y. The percentages aged <35, 35–44, 45–54, 55–64, and 65 y or older at diagnosis were 5%, 10%, 22%, 32%, and 31%, respectively. Among controls the average height was 162.7 (standard deviation, 7.0) cm, and the average body mass index was 25.0 (standard deviation, 4.9) kg/m^2^.

**Figure 1:**
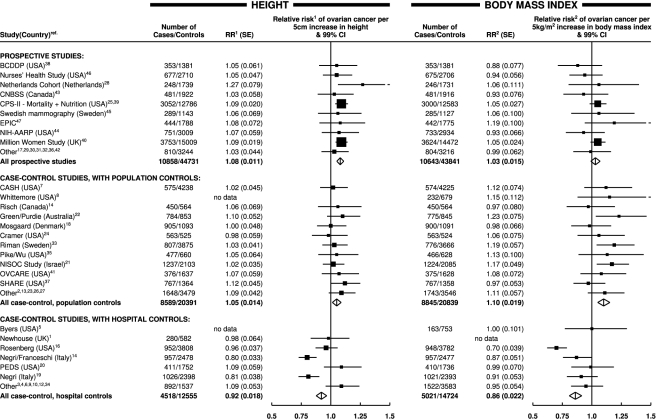
Relative risk of ovarian cancer in relation to height and body mass index by study. Relative risk^1^ (RR^1^) is stratified by study, age at diagnosis, parity, menopausal status/hysterectomy, body mass index, duration of oral contraceptive use, and ever use of hormone therapy. Relative risk^2^ (RR^2^) is stratified by study, age at diagnosis, parity, menopausal status/hysterectomy, height, duration of oral contraceptive use, and ever use of hormone therapy. SE, standard error.

Ovarian cancer risk increased significantly both with increasing height and with increasing body mass index (*p*<0.001 for each). However, there was highly significant variation in the findings by study design (*p*_heterogeneity_<0.001 for each); this is illustrated in Figure 1, which shows study-specific relative risks of ovarian cancer risk per 5 cm increase in height and per 5 kg/m^2^ increase in body mass index, grouped by study design. Studies contributing relatively small amounts of statistical information (where the reciprocal of var[log relative risk] is less than 30) are included in the “other” category for the relevant study design. The variation by study design is largely due to the qualitatively different results for case–control studies with hospital controls compared to results for case–control studies with population controls or for prospective studies. The possibility that some of the hospital controls were women with conditions affected by height and body mass index cannot be excluded, and so studies with hospital controls are excluded in subsequent analyses (see Discussion).

### Height

The taller women were, the greater was their risk of ovarian cancer (Table 2). The adjusted relative risk for ovarian cancer was 1.07 (95% CI, 1.05–1.09, *p*<0.001) for every 5 cm increase in height. Analyses were stratified by study, age, parity, menopausal status, hysterectomy, oral contraceptive use, use of hormone therapy for the menopause, and body mass index. It can be seen in Table 2 that, without stratification for body mass index, the relative risk estimates associated with increasing height were very slightly lower, by less than 1%, than they were with such stratification. We also assessed the effect of further adjustment for seven other potential confounding factors: ethnic group, education, age at first birth, family history of ovarian or breast cancer, age at menarche, alcohol use, and tobacco consumption. The estimates of relative risk were altered by less than 1% by additional adjustment for individual factors, and simultaneous adjustment for all seven factors did not alter the estimate.

**Table 2:** Relative risk of ovarian cancer in relation to height, weight, and body mass index.

Factor	Cases/Controls	Relative Risk^a^ (99% g-s CI)	Relative Risk^b^ (99% g-s CI)
**Height (cm) (mean)**			
<160 (154.8)	5,221/18,334	1.00 (0.95–1.05)	1.00 (0.95–1.05)
160– (161.7)	5,678/19,231	1.09 (1.05–1.14)	1.10 (1.05–1.16)
165– (166.5)	4,961/16,498	1.14 (1.09–1.20)	1.15 (1.10–1.21)
170+ (172.7)	3,587/11,059	1.24 (1.17–1.32)	1.27 (1.20–1.35)
**Weight (kg) (mean)**			
<60 (54.1)	5,870/19,904	1.00 (0.96–1.05)	1.00 (0.95–1.05)
60–69 (64.2)	6,379/22,197	1.04 (1.00–1.08)	1.02 (0.97–1.06)
70–79 (73.7)	3,939/13,183	1.11 (1.05–1.17)	1.07 (1.02–1.14)
80+ (90.3)	3,114/9,128	1.24 (1.17–1.32)	1.18 (1.10–1.26)
**Body mass index (kg/m^2^) (mean)**			
<22.5 (20.6)	6,299/21,009	1.00 (0.96–1.05)	1.00 (0.95–1.05)
22.5– (23.5)	4,024/13,554	1.04 (0.99–1.10)	1.05 (1.00–1.11)
25– (25.9)	4,351/14,790	1.07 (1.02–1.12)	1.08 (1.02–1.13)
27.5– (28.4)	1,771/5,892	1.07 (0.99–1.16)	1.07 (0.99–1.17)
30+ (33.6)	3,043/9,435	1.12 (1.05–1.19)	1.13 (1.06–1.20)

Test for trend: height, *p*<0.001; weight, *p*<0.001; body mass index, *p*<0.001. Studies with hospital controls are excluded.

a Relative risk stratified by study, age at diagnosis, parity, menopausal status/hysterectomy, duration of oral contraceptive use, and ever use of menopausal hormone therapy.

b Relative risk stratified as for the previous column, with additional stratification by body mass index (for height) and by height (for weight and body mass index).

**Figure 2:**
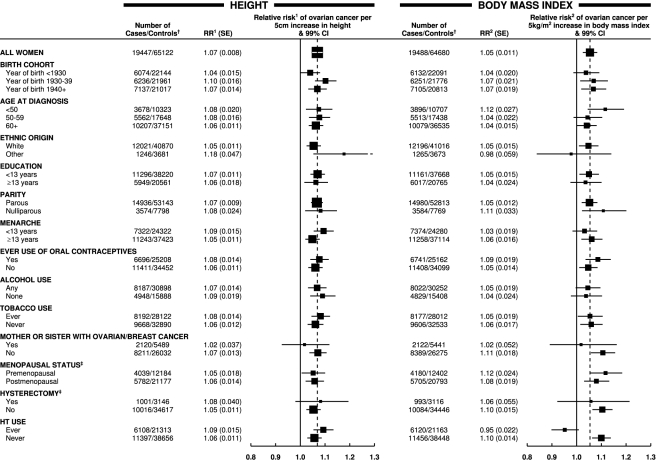
Relative risk of ovarian cancer in relation to height and BMI in various subgroups of women. Relative risk^1^ (RR^1^) is stratified by study, age at diagnosis, parity, menopausal status/hysterectomy, body mass index, duration of oral contraceptive use, and ever use of hormone therapy (HT). Relative risk^2^ (RR^2^) is stratified by study, age at diagnosis, parity, menopausal status/hysterectomy, height, duration of oral contraceptive use, and ever use of hormone therapy. ^†^ numbers do not always add to the total because of missing values; ^‡^ never-user of hormone therapy. Case–control studies with hospital controls are excluded. The dotted line represents the overall result for all women. SE, standard error.

**Figure 3:**
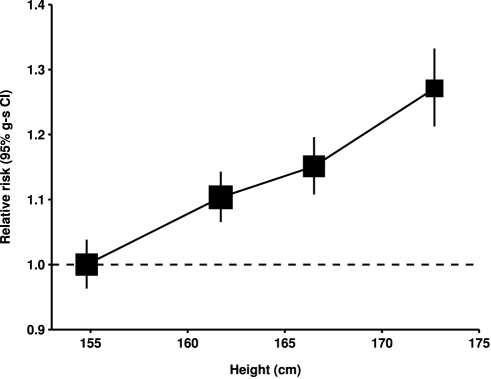
Relative risk of ovarian cancer by height. Relative risk compared to women with height <160 cm and stratified by study, age at diagnosis, parity, menopausal status/hysterectomy, body mass index, duration of oral contraceptive use, and ever use of hormone therapy. Relative risk estimates are plotted against the mean height in each category (<160, 160–164, 165–169, and 170+ cm). Case–control studies with hospital controls are excluded.

The magnitude of the increase in the relative risk of ovarian cancer with increasing height did not vary substantially by age, year of birth, education, age at menarche, parity, use of oral contraceptives, menopausal status, use of menopausal hormone therapy, hysterectomy, smoking, alcohol consumption, or having first degree relatives with breast or ovarian cancer (Figure 2). The trend appeared to be somewhat greater in non-white than in white women, but the difference was of borderline significance (*p*_heterogeneity_ = 0.02), and, given the large number of comparisons made, it may well be due to chance. There was no significant heterogeneity in the relationship between height and ovarian cancer risk between prospective studies and case–control studies with population controls (Figure 1; *p*_heterogeneity_ = 0.07). Since the associations did not vary materially across the various subgroups studied and additional adjustment for other potential confounders had little effect, the overall relationship between height and the relative risk of ovarian cancer can be summarized as shown in Figure 3, where the relative risks in each category of height are plotted against the mean height in that category.

### Weight and Body Mass Index

The heavier and the more obese women were, the greater was their risk of ovarian cancer (Table 2). These results were obtained after all data were stratified by age, study, parity, menopausal status, hysterectomy, oral contraceptive use, use of hormone therapy for the menopause, and height. As expected, adjustment for height reduced the relative risks of ovarian cancer associated with increasing weight but had little effect on the relative risks associated with increasing body mass index (Table 2). Since the association between weight and ovarian cancer risk is dependent on height, but the association with body mass index is not, subsequent analyses focus on body mass index.

The magnitude of the increase in the relative risk of ovarian cancer with increasing body mass index did not vary significantly by women's age, year of birth, ethnic group, education, age at menarche, parity, use of oral contraceptives, menopausal status, hysterectomy, alcohol and tobacco use, or family history of breast or ovarian cancer (Figure 2). However, use of hormone therapy for the menopause had a substantial effect on the association (*p*_heterogeneity_<0.001). The relative risk of ovarian cancer increased with increasing body mass index among never-users of menopausal hormone therapy, but not among ever-users (relative risk per 5 kg/m^2^ increase in body mass index of 1.10 [95% CI, 1.07–1.13], *p*<0.001; and 0.95 [95% CI, 0.92–0.99], *p* = 0.02, respectively). Given that almost 60 subgroup-specific relative risks are shown in Figure 2, the *p*-value of 0.02 for users of hormone therapy is of borderline significance, whereas the *p*-values of <0.001 for the trend in non-users and for the difference in the trends between hormone users and non-users is unlikely to be due to chance. Such differences were observed both in prospective studies (1.08 [95% CI, 1.04–1.12] versus 0.94 [95% CI, 0.90–0.99]; *p*_heterogeneity_<0.001) and in case–control studies with population controls (1.13 [95% CI, 1.08–1.18] versus 0.99 [95% CI, 0.91–1.08]; *p*_heterogeneity_ = 0.008) and did not differ when analyses were restricted to postmenopausal women.

**Figure 4:**
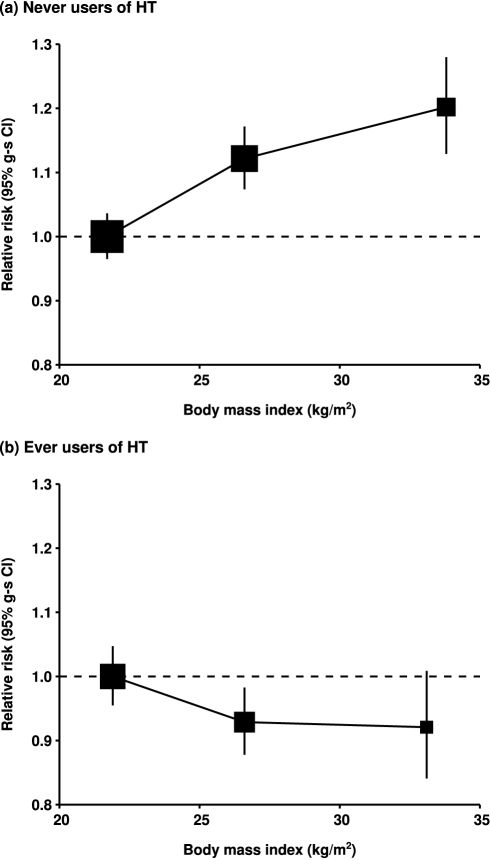
Relative risk of ovarian cancer by body mass index. Relative risk in (A) never-users of hormone therapy (HT) and (B) ever-users of hormone therapy, taking women with a body mass index of <25 kg/m^2^ in each group as the baseline (relative risk = 1.0), and stratified by study, age at diagnosis, parity, height, and duration of oral contraceptive use. Results for never-users of hormone therapy are additionally stratified by menopausal status/hysterectomy, and results for ever-users of hormone therapy are restricted to postmenopausal women. Relative risk estimates are plotted against the mean body mass index in each category (<25, 25–29, and 30+ kg/m^2^). Case–control studies with hospital controls are excluded.

Results in prospective studies were not significantly different when the first 4 y of follow-up were excluded. The risk estimates associated with body mass index in ever-users and never-users of hormone therapy changed by less than 2% after further adjustment for seven other potential confounding factors: ethnic group, education, age at first birth, family history of ovarian or breast cancer, age at menarche, and alcohol and tobacco consumption, and simultaneous adjustment for all these factors also affected the relative risk estimate by less than 2%. Since factors other than hormone use did not have a major effect on the relationship between ovarian cancer and body mass index, the main results are summarized in Figure 4 separately for never-users and ever-users of menopausal hormone therapy. The category-specific relative risks for ovarian cancer are plotted against the mean body mass index in each category.

### Tumour Histology

**Figure 5:**
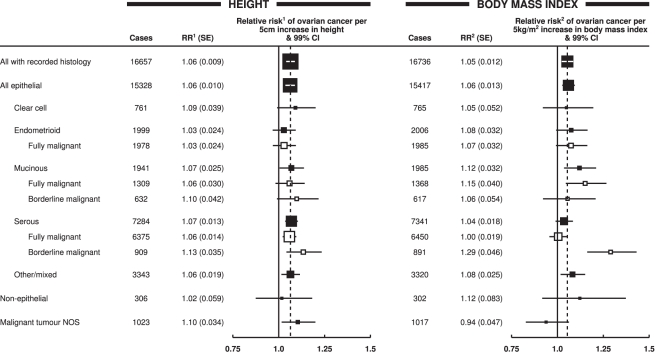
Relative risk of ovarian cancer by tumour histology. Relative risk^1^ (RR^1^) is stratified by study, age at diagnosis, parity, menopausal status/hysterectomy, body mass index, duration of oral contraceptive use, and ever use of hormone therapy. Relative risk^2^ (RR^2^) is stratified by study, age at diagnosis, parity, menopausal status/hysterectomy, height, duration of oral contraceptive use, and ever use of hormone therapy. Case–control studies with hospital controls are excluded. The dotted line represents the overall result for all women with recorded histology. NOS, not otherwise specified; SE, standard error.

Data on tumour histology was available for 17,039 (68%) women with ovarian cancer included in the main analyses. The effects of increasing height and increasing body mass index did not differ significantly between epithelial and non-epithelial tumours or between clear cell, endometrioid, mucinous, or serous histology (Figure 5). However, when the data for mucinous and serous tumours were subdivided into whether they were of only borderline malignancy or were fully malignant, the trend with increasing body mass index was considerably greater for borderline serous tumours than for fully malignant serous tumours (*p*_heterogeneity_<0.001). Borderline malignant serous tumours make up about 5% of all the ovarian tumours included here. There were too few borderline malignant endometrioid tumours to examine separately. When analyses were restricted to never-users of menopausal hormone therapy, the associations with body mass index shown in Figure 5 did not change materially. The stronger association with borderline than with fully malignant serous tumours remained (relative risks per 5 kg/m^2^ increase in body mass index of 1.33 [95% CI, 1.20–1.47] and 1.04 [95% CI ,1.00–1,09], respectively, *p*_heterogeneity_<0.001); for the other tumour types the corresponding relative risks among never-users of hormone therapy were 1.05 (95% CI, 0.93–1.17) for clear cell, 1.10 (95% CI, 1.02–1.19) for endometrioid, and 1.14 (95% CI, 1.06–1.22) for mucinous tumours.

### Public Health Implications

Based on the findings here, a 1 cm increase in height is associated with a relative risk of ovarian cancer of 1.014, and a 1 kg/m^2^ increase in body mass index with a relative risk of 1.019, in never-users of hormone therapy. In high-income countries, where average height has increased by about 1 cm per decade [58] and average body mass index by about 1 kg/m^2^ per decade [59], the associated increase in ovarian cancer incidence would be 3% (1.019×1.014 = 1.03) per decade in never-users of hormone therapy, if all other factors relevant for the disease remained constant.

## Discussion

Our collaboration has brought together and re-analysed individual data from 47 studies on ovarian cancer risk associated with adult height, weight, and body mass index, that is, most of the available epidemiological information worldwide. Collectively, the findings show a highly significant increase in the risk of ovarian cancer with increasing values for each of the anthropometric variables examined. The increase in ovarian cancer risk with increasing height and with increasing body mass index did not vary materially by women's age, year of birth, ethnicity, education, age at menarche, parity, family history of ovarian or breast cancer, use of oral contraceptives, menopausal status, hysterectomy, or consumption of alcohol and tobacco. However use of hormone therapy for the menopause attenuated the relationship with body mass index, since an increase in ovarian cancer risk with increasing adiposity was found only in never-users of such therapy. The trends with increasing height and body mass index were broadly similar across the common histological subtypes of ovarian cancer, except for serous tumours of borderline malignancy, which comprise 5% of the total, where the increase in risk with increasing body mass index was considerably greater than for the other tumour subtypes.

An advantage of seeking to review all epidemiological studies of ovarian cancer with relevant information on body size, published and unpublished, is that this helps avoid unduly selective emphasis on published results or just on some studies. Three eligible studies that did not contribute data to these analyses but that had published on ovarian cancer risk associated with height and/or body mass index, [48]–[51], and some small cohorts that contributed to a fourth study [52], together contain less than 10% as many women with ovarian cancer as are included here, and as their findings do not differ materially from those reported here, failure to include them will not have materially altered the relationships reported here. Only about half the eligible studies had published on body size and ovarian cancer risk. Few of these studies published separate results by use of hormone therapy, and so reviews based solely on published findings on the relation between body mass index and ovarian cancer risk could be misleading. The total number of studies that did not meet the eligibility criteria for this collaboration is unknown, as details of such studies have not been routinely collected over the 15 y since the collaboration began. Furthermore, despite extensive efforts to identify all eligible studies with unpublished results, it is clearly impossible to guarantee that others do not exist. It is also not possible to have completely up-to-date information from continuing prospective studies that are accumulating data beyond the time when information was contributed to this collaboration. Unpublished results from known continuing prospective studies may contain at least another 5% as many women with ovarian cancer as are included here, but there is no good reason to expect that over the next few years inclusion of additional data from such studies will materially alter the evidence that is already available.

Results from case–control studies that used hospital controls differ qualitatively and significantly from those with other study designs. It seems unlikely that these differences are due to the retrospective reporting of height and/or weight, since the results differ substantially between the retrospective studies that used hospital controls and the retrospective studies that used population controls. While other factors might also be relevant, many of the retrospective studies with hospital controls were designed to examine the effects of hormonal factors on ovarian cancer risk, and controls were often recruited from orthopaedic wards, where the most common reason for admission among middle-aged women is for hip and knee replacement surgery, and the risk of these conditions increases markedly with increasing height and increasing body mass index [60]. Using such controls to study the role of anthropometric factors in ovarian cancer would dilute, and could even reverse, any association. Since insufficient information was provided centrally on diagnoses among controls, the possibility of such biases could not be excluded, and studies with hospital controls were omitted from the main analyses. Nevertheless, to ensure that the totality of the epidemiological information is published, details of those studies are given in Table 1 and in Figure 1.

In prospective studies, height and weight were generally recorded at the time that women were recruited into the cohort, and in case–control studies, women were generally asked about their height and weight some 1–5 y before the diagnosis of ovarian cancer (for cases) and at an equivalent time for controls. In the case–control studies with population controls, the retrospective reporting of height and weight may have been influenced by the cases' knowledge that they had ovarian cancer, but the similarity of the findings in such studies and in those with prospective recording of anthropometric factors suggests that this is probably not a serious problem here. Nonetheless, there is still likely to be some measurement error, not only in retrospective self-reported data but also with measurements made at entry into a prospective study, because women's weight (and, to a lesser extent, their height) may change over time. Non-differential misclassification of these variables would dilute estimates of relative risk, although the effects are likely to be comparatively small [61].

The observed association between ovarian cancer and height did not differ significantly between prospective studies and retrospective studies with population controls, nor did it vary by the 12 personal characteristics of the women that were examined (Figure 2). Variation in height reflects genetic and environmental influences, acting mostly in the first 20 or so years of life, with the role of environmental factors, including childhood nutrition and infections, believed to predominate [62]. The observed relationship may reflect the biological influence of factors associated with adult height that are not yet well characterized or understood, such as the number of cells at risk of developing into cancer [63] or variation in levels of circulating growth factors, such as insulin-like growth factor-1 [64].

The association between ovarian cancer and body mass index is seen across all subgroups of women studied, except ever-users of menopausal hormone therapy. The relevance of the finding in users of menopausal hormones will be examined in a future report from this collaboration, where the direct relationship between ovarian cancer risk and use of menopausal hormone therapy will be examined, and the potential modification of any effect of hormone therapy by women's adiposity and other factors will be explored. Body mass index is in general a good measure of adiposity, although women with the same value for the index may vary in the relative contribution from fat and from muscle. The finding that use of hormone therapy largely eliminates the relationship between body mass index and ovarian cancer risk suggests that endogenous oestrogens may be relevant, at least among postmenopausal women: the well-characterized association between circulating oestrogen levels and increasing adiposity in postmenopausal women who do not use hormonal therapies is likely to be altered among users of these therapies [65]. It is unknown why the association with body mass index was greater for borderline malignant serous tumours than for other ovarian tumour types. Borderline malignant serous tumours appear to differ from fully malignant serous tumours in certain mitochondrial DNA sequences, but the relevance of these genetic differences to the observed associations with adiposity is unclear [66].

Among women in high-income countries, average height has increased by about 1 cm per decade and average body mass index has increased by about 1 kg/m^2^ per decade in the generations of women now developing ovarian cancer [58],[59]. As an illustration of the public health consequences of such changes in height and weight, these findings suggest an associated increase in ovarian cancer incidence of 3% per decade if all other factors relevant for ovarian cancer remained constant.

## Supporting Information

Text S1 **PRISMA 2009 checklist.**
(DOC)

## Abbreviations

CI: confidence interval
g-s CI: group-specific confidence interval
